# Cost-Effectiveness Analysis of Direct Oral Anticoagulants Versus Vitamin K Antagonists for Venous Thromboembolism in China 

**DOI:** 10.3389/fphar.2021.716224

**Published:** 2021-10-20

**Authors:** Ke-Xin Sun, Bin Cui, Shan-Shan Cao, Qi-Xiang Huang, Ru-Yi Xia, Wen-Jun Wang, Jing-Wen Wang, Feng Yu, Yi Ding

**Affiliations:** ^1^ Department of Pharmacy, Xijing Hospital, Fourth Military Medical University, Xi’an, China; ^2^ School of Basic Medicine and Clinical Pharmacy, China Pharmaceutical University, Nanjing, China; ^3^ Department of Epidemiology and Biostatistics, School of Public Health, Xi’an Jiaotong University Health Science Center, Xi’an, China

**Keywords:** VTE, DOAC, CEA = cost-effectiveness analysis, China, LMWH (low molecular weight heparin)

## Abstract

**Background:** The drug therapy of venous thromboembolism (VTE) presents a significant economic burden to the health-care system in low- and middle-income countries. To understand which anticoagulation therapy is most cost-effective for clinical decision-making , the cost-effectiveness of apixaban (API) versus rivaroxaban (RIV), dabigatran (DAB), and low molecular weight heparin (LMWH), followed by vitamin K antagonist (VKA), in the treatment of VTE in China was assessed.

**Methods:** To access the quality-adjusted life-years (QALYs) and incremental cost-effectiveness ratios (ICERs), a long-term cost-effectiveness analysis was constructed using a Markov model with 5 health states. The Markov model was developed using patient data collected from the Xijing Hospital from January 1, 2016 to January 1, 2021. The time horizon was set at 30 years, and a 6-month cycle length was used in the model. Costs and ICERs were reported in 2020 U.S. dollars. One-way sensitivity analysis and probabilistic sensitivity analysis (PSA) were used to test the uncertainties. A Chinese health-care system perspective was used.

**Results:** In the base case, the data of 231 VTE patients were calculated in the base case analysis retrospectively. The RIV group resulted in a mean VTE attributable to 95% effective treatment. API, DAB, and VKA have a negative ICER (−187017.543, −284,674.922, and −9,283.339, respectively) and were absolutely dominated. The Markov model results confirmed this observation. The ICER of the API and RIV was negative (−216176.977), which belongs to the absolute inferiority scheme, and the ICER value of the DAB and VKA versus RIV was positive (110,577.872 and 836,846.343). Since the ICER of DAB and VKA exceeds the threshold, RIV therapy was likely to be the best choice for the treatment of VTE within the acceptable threshold range. The results of the sensitivity analysis revealed that the model output varied mostly with the cost in the DAB on-treatment therapy. In a probabilistic sensitivity analysis of 1,000 patients for 30 years, RIV has 100% probability of being cost-effective compared with other regimens when the WTP is $10973 per QALY. When WTP exceeded $148,000, DAB was more cost-effective than RIV.

**Conclusions:** Compared with LMWH + VKA and API, the results proved that RIV may be the most cost-effective treatment for VTE patients in China. Our findings could be helpful for physicians in clinical decision-making to select the appropriate treatment option for VTE.

## Highlights

“What is already known about this subject”:

• VTE is a significant cause of morbidity and mortality worldwide and is associated with a substantial economic burden.
• The most cost-effective anticoagulant treatment option for VTE remains controversial.

“What this study adds”:

• RIV is likely to be considered a cost-effective or cost-saving strategy for VTE patients in China.
• When the willingness to pay exceeded $148,000, DAB was more cost-effective than RIV.
• This study could support the decision-making of stakeholders in China, including hospitals, payers, and physicians.

## Introduction

Venous thromboembolism (VTE), including deep vein thrombosis (DVT) and pulmonary embolism (PE), is a common clinical peripheral vascular disease and disproportionately impacts adults worldwide (Gould et al., 2012; Nemeth et al., 2019; Chopard et al., 2020). An estimated one in 12 people older than 45 years will be at risk of VTE (Cushman et al., 2020). The mortality of VTE can be as high as 10–30% within one month in high-risk patients (Renner and Barnes, 2020). The economic burden caused by VTE can reach one billion or even tens of billions of dollars each year in European countries (Di Nisio et al., 2016; Barco et al., 2020). Compared with Western countries, Asian populations are known to have lower VTE incidences, which are estimated to be approximately 15–20% of the level recorded in Western countries (Raskob et al., 2014). However, the detection rate of VTE in the Asian population has increased greatly in recent years with the improvement of diagnostic levels and diagnostic awareness (Lee et al., 2017). Especially, the hospitalization rate in China is, indeed, increasing from 3.2 to 17.5 per 100,000 population (Angchaisuksiri et al., 2021) due to the increase in the age of the population, the incidence of cancer, and the number of operations (Zhai et al., 2019). Moreover, considering the risk of death from the disease, patients often stay in the hospital for longer periods, which will impose a greater social and economic burden on the health-care system (Zhang et al., 2019).

Current guidelines (Kakkos et al., 2020) for the management of VTE in 2021 recommended the use of direct oral anticoagulants (DOACs) over vitamin K antagonists (VKAs) for the initial and secondary treatment of VTE. Low molecular weight heparin (LMWH) overlapped with VKAs has been considered a standard treatment for many years. Recently, DOACs have been increasing in popularity and availability, including apixaban (API), rivaroxaban (RIV), and dabigatran (DAB) (Ortel et al., 2020). A 2014 review (Wu et al., 2014a, b) comparing the results of five randomized clinical trials has identified that DOACs have similar efficacy to VKA in the treatment of VTE but significantly reduce the risk of major bleeding (MB). Moreover, DOACs do not require monitoring, take effect quickly, and avoid bridging with load and LMWH (López-López et al., 2017). However, the drug acquisition cost of DOACs was higher than that of VKA (US$39.47/2.5 mg versus 0.18/2.5 mg) according to data from the IQVIA China Hospital Pharmaceutical Audit Database. Although Chinese medical insurance can only partially reimburse the cost of DOACs (70–80%), it is limited to patients with non-valvular atrial fibrillation and lower extremity joint replacement surgery.

Up to now, the National Institute for Health and Care Excellence (NICE) guideline (Howard and Hughes, 2013) team pointed out that the most cost-effective therapy should be treated with caution and is still controversial. Lanitis (Lanitis et al., 2016) conducted a pharmacoeconomic analysis based on the AMPILIFY (Li:QY et al., 2015) clinical trial in 2016. The results showed that API is a cost-effective therapeutic option versus the standard therapy for VTE. Nevertheless, the NICE constructed a cost–utility analysis from an NHS/personal social perspective, which showed that the costs were partially offset by fewer surveillance visits and lower resource usage associated with managing major bleeding events (Schulman et al., 2020). The economic research conducted in China has also differed results. One cost-effectiveness (Xiaoyu et al., 2016) strategy based on two RCTs indicated that the use of API for VTE does not represent a good value for the cost at the acceptable threshold in China. A 2020 literature (Wang Sheng-xiang et al., 2020) whose probability was determined by meta-analysis showed that RIV had economic advantages over standard therapies and other DOACs. It can be seen that most studies are based on literature research or RCT evidence. However, RCT evidence has strict inclusion and exclusion criteria, which makes it difficult to extrapolate the research results to clinical practice (Sanson-Fisher et al., 2007). In addition, the existing economic evaluations mainly focused on the comparison of one DOAC versus VKA or different DOACs. Nonetheless, only comparing the results of two interventions once may not help clinicians to choose the best option when several treatment options coexist.

The objective of this study is to compare the cost-effectiveness of four regimens at the same time both in the short-term hospitalization period and long-term Markov model in VTE from the Chinese health-care system’s perspective. In this way, the results of this study will provide for clinical decision-making in VTE patients and the optimization of health-care resource allocation.

## Methods

The patient data were retrospectively obtained from the EMR database of VTE patients at Xijing Hospital in Xi’an, China, from January 1, 2016 to January 1, 2021. The study was approved by the Xijing Hospital Institutional Review Board (KY20212011-C-1). The guideline checklist reported in the Consolidated Health Economic Evaluation Reporting Standards (CHEERS) was followed (Kong et al., 2009; Weinstein et al., 2010; Husereau et al., 2013).

### Patients and Intervention

**Inclusion criteria:** 1) Patients diagnosed with VTE according to the European Society for Vascular Surgery (ESVS) 2021 Guidelines; 2) Anticoagulant drugs used by patients are one of the following: API, RIV, DAB, and LMAH + VKA; 3) age>18 years old; and 4) the data and medical records are complete.

**Exclusion criteria:** 1) Patients have anticoagulation contraindications; 2) patients who have not completed standardized treatment in this hospital and are discharged automatically; and 3) drug abuse or mental illness that may interfere with treatment.

### Usage and Dosage of Drugs

The dose and course of treatment are determined according to the guidelines recommended (Kakkos et al., 2020; Renner and Barnes, 2020): anticoagulation therapy strategies should be conceptualized in 3 phases: initial management (5–21 days), primary treatment (3–6 months), and secondary prevention (beyond 3–6 months). Based on the recommendation, rivaroxaban was prescribed at a dose of 15mg, BID for 21 days, followed by 20 mg once daily until 6 months. Apixaban treatment consisted of a 7-day course of 10 mg twice a day, followed by 5 mg twice a day. Patients with dabigatran therapy take LMWH 0.6 ml/6000 IU, BID from day 1 to 5, then stop and use dabigatran 150 mg twice daily. For patients who will be transitioning to warfarin, LMWH is commonly used in the primary treatment phase, followed by 5 mg warfarin daily adjusted to the target INR 2.0–3.0.

Adverse reactions such as MB, clinically relevant non-major bleeding (CRNMB), and death in the patient were observed and recorded within the hospitalization period. Major bleeding was defined as clinically significant and associated with a reduction in hemoglobin levels of at least 20 g/L, or bleeding occurring in a critical site (Schulman et al., 2005). CRNMB was defined as any significant bleeding not fitting the criteria for major bleeding (Kaatz et al., 2015). Means and standard deviations (SD) of all types of resource utilizations were calculated.

### Model Structure

**FIGURE 1:**
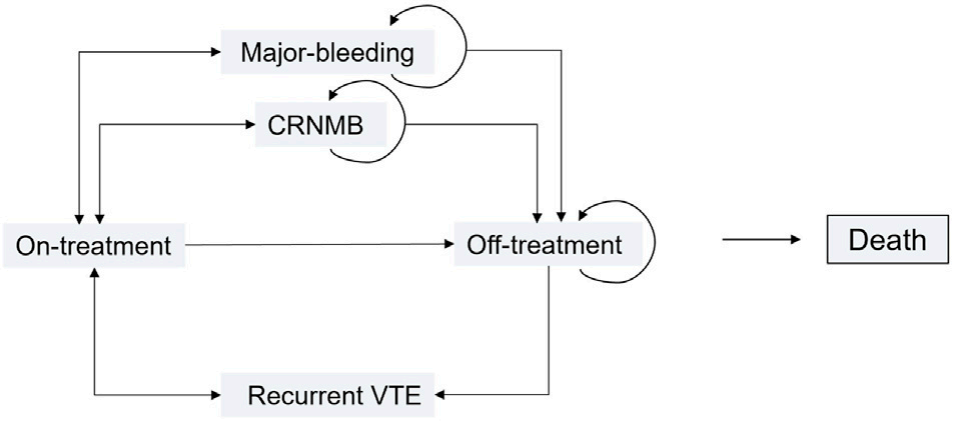
Markov state transition model.

A long-run Markov model was developed to evaluate the cost-effectiveness analysis, which estimated the costs and health outcomes of treating VTE using DOACs in patients. The Markov state transition model is shown in Figure 1. The process included six discrete health states: VTE on-treatment, VTE off-treatment, recurrent VTE, MB, CRNMB, and the absorbing state of death. Patients entered the model with “on-treatment” status after diagnosis of VTE. The initial assessment and treatment differences by physicians and providers were ignored, assuming that these costs were the same between groups. Only costs after treatment were assessed. A cohort of individuals aged 59 years was followed in the model which was calculated from the base case. Off-treatment refers to stopping treatment for any reason after the individual expects the treatment to end. Patients can progress from any other health state than the CRNMB state to the death health state. The same patient can only experience one of the predicted states or remain unchanged in the current health state. Because the American Society of Hematology 2020 guidelines recommended (Ortel et al., 2020) that primary treatment continues anticoagulant therapy for 3–6 months for the treatment of VTE, we set the cycle length to be 6 months. The time horizon was set to be 30 years. To calculate the dosage of LMWH and warfarin, we assumed a typical patient weighed 60 kg.

### Model Input

All model parameters collected in this study mainly consisted of cost, transition probability, and health utility value (Table 1). The clinical effects and cost parameters were quoted from electronic medical records (EMR) at Xijing Hospital. Based on previous studies, the transition probabilities between different health states were estimated. Some other outcome probabilities and utilization data were obtained from the literature review. The following formula (Briggs and Sculpher, 1998; Petitti, 2002) was used to calculate the transition probabilities of one cycle: r = -[In(1–P_1_)]/t_1_; P_2_ = 1–exp (-rt_2_); r represents the transient probability, and P_1_ and P_2_ represent the transition probability for a given cycle t_1_ and t_2_, respectively. Moreover, this study assumed the blank data by asking for expert advice.

**TABLE 1:** Model inputs.

Cost in different states	Base case	Range tested	Distribution	Source
Recurrent VTE	3,853	2,697–5,009	Gamma	LI
MB	3,834	2,684–4,984	Gamma	Wu(Wu et al.)
CRNMB	8.25	5.77–10.72	Gamma	Wu(Wu et al.)
Warfarin monitoring (per time)	10.98	7.69–14.27	Gamma	EMR
Utilities				
VTE on-treatment	0.94	0.75–1.00	Beta	Mccullagh (Mccullagh et al., 2012)
Recurrent VTE	0.76	0.57–0.95	Beta	Uniform
MB	0.55	0.15–0.86	Beta	Hogg (Hogg et al., 2013)
CRNMB	0.61	0.68–0.51	Beta	Locadia (Locadia et al., 2004)
Death	0.00	-	Beta	Definition
VTE off-treatment	0.75	0.45–0.91	Beta	-
API	−0.0020	0.000–0.0060	Beta	Gage(Gage et al., 1996)
VKA	−0.0130	0.000–0.0047	Beta	Gage(Gage et al., 1996)
RIV	−0.002	0.000–0.006	Beta	Gage(Gage et al., 1996)
DAB	−0.002	0.000–0.005	Beta	Gage(Gage et al., 1996)
Cost of drugs				
API	5,877.399	639.764–14,326.537	Gamma	EMR
RIV	3,072.136	465.279–18,391.693	Gamma	EMR
DAB	3,926.160	970.546–13,152.974	Gamma	EMR
VKA	4,325.386	612.487–10,287.356	Gamma	EMR

VTE, venous thromboembolism; MB, major bleeding; CRNMB, clinically relevant non-major bleeding; API, apixaban; VKA, vitamin K antagonist; RIV, rivaroxaban; DAB, dabigatran; EMR, electronic medical records.

For comparability, all costs were expressed in U.S. dollars for the 2021 reference year in this study. Chinese yuan (CNY) was converted into U.S. dollars by using the following exchange rate:1US$ = CNY6.46 (2020). From the Chinese health-care perspective and considering the proportion of direct medical costs and direct non-medical costs to direct costs, the cost of this study is proposed as direct medical costs. Utility level values for other health states were obtained from the literature search. According to the current pharmacoeconomic guidelines in China (Liu et al., 2015; Paulden et al., 2017), the discount rate used in this study is 5% (0–8%).

### Outcomes

The primary result is the incremental cost-effectiveness ratio (ICER) to evaluate and select multiple programs, presented in costs per quality-adjusted life year (QALY). In this study, the lowest cost-effectiveness ratio (CER) treatment therapy in each group was used as the control. The ICER between other plans and the control treatment therapy was calculated separately to analyze the choice of the most cost-effective therapy. According to the World Health Organization (WHO, 2003) guidelines, if the additional cost of switching to a new treatment plan to obtain an additional effect is less than three times the country-specific per capita gross domestic product (GDP), then the treatment plan is considered acceptable by the patient. It was regarded as cost-effective if the ICER was less than per capita GDP. Therefore, this study sets the value that people will pay as one to three times of GDP (10,973–32,921$/year) in 2020 (Guo, 2020). To determine the most cost-effective option using net life years or QALY gained, 1,000 iterations of Monte Carlo simulations were performed to construct the acceptability curve of the therapies.

### Statistical Analysis

Measurement data were expressed as mean ± standard deviation. Model development, implementation, and analysis were performed using TreeAge Pro (TreeAge Software, Inc., Williamstown, MA, United States) for queue simulation and sensitivity analysis. The Markov model cycle length was set as 6 months.

The Markov model parameters in this study are derived from the EMR database. Due to the differences in research design, data statistics, and research conditions, sensitivity analysis was carried out to correct the model (Naimark et al., 2008). One-way sensitivity analysis and probabilistic sensitivity analysis were conducted to access the uncertainty in the model. The study used 95% CIs as the upper and lower limits of the health state utilities. A range of ±20% of the base-case value was used for costs.

The results of one-way sensitivity analysis were displayed in the form of tornado diagrams. The variables that have the greatest impact on the collaboration results were drawn in turn. By defining the distribution for key parameters (utilities were defined as beta distribution and gamma distribution for costs), probablistic sensitivity analysis (PSA) was performed to assess the overall impact of the model’s uncertainty. Monte Carlo simulation was performed 1,000 times to analyze multiple uncertain factors, which are represented by the cost-effectiveness acceptable curve and ICER scatter diagram. The results of the PSA were described as scatterplots.

## Results

### Base-Case Analysis

A total of 551 patients with VTE were collected. Two hundred one patients were excluded because of the incomplete data. Ninety-eight patients who were not on a single drug medication and 21 patients who dropped out of the study were excluded. The data were retrospectively collected from the medical records of 231 patients who received four therapies. The characteristics of the patients are shown in Table 2. According to the hospitalization records of the EMR (Table 3), the effective rate of treatment and the incidence of the adverse reactions were calculated as shown in Table 4. In this primarily included cohort, patients in the VKA group were younger but had higher unfavorable therapy rate. The drug acquisition cost of API was higher than that of others (US$39.47/2.5 mg), and VKA was the lowest (US$0.18/2.5 mg) (Supplementary Table S1). However, the monitoring cost and the cost of blood tests with intravenous injections which were included the therapy fee (US$2056.08), made the total cost of VKA not the lowest of the four treatment options.

**TABLE 2:** Comparison of the baseline characteristics of the four groups.

Characteristic	Apixaban (N = 50)	Rivaroxaban (N = 110)	Dabigatran (N = 21)	LMWH/VKA (N = 50)
Age (yr)				
Mean	58 ± 16.3	62 ± 11.5	64 ± 14.6	53 ± 13.6
Range	24–94	29–87	32–92	19–75
Age category (years), n (%)				
<75	78	86.4	81.0	94.0
≥75	22	13.6	19.0	6.0
Female sex, no. (%)	50.0	42.7	52.4	50.0
Weight (kg)				
Mean	65 ± 11.8	77 ± 11.1	65 ± 10.2	66 ± 1.0
Range	40–90	46–95	50–80	45–96
BMI (kg/m^2^)	23 ± 3.3	23 ± 6.4	25 ± 3.2	24 ± 3.0
Length of hospital stay				
Mean	8 ± 6.2	9 ± 7.6	11 ± 10.1	10 ± 8.4
Range	1–29	1–51	2–48	3–56
Diabetes mellitus, no. (%)	2.0	8.2	19.0	2.0
Hypertension, no. (%)	18.0	33.6	19.0	10.0
Type of index event, no. (%)				
DVT only	98.0	30.0	80.9	32.0
PE only	0.0	29.1	19.1	38.0
Both DVT and PE	2.0	40.9	0.0	30.0

BMI: body mass index, API, apixaban; RIV, rivaroxaban; DAB, dabigatran; LMWH/VKA, low molecular weight heparin followed by vitamin K antagonist. DVT, deep vein thrombosis; PE, pulmonary embolism.

**TABLE 3:** Results of EMR data for hospitalization costs.

	Laboratory costs	Bed costs	Operation costs	Nursing costs	Radiation costs	Examination costs	Treatment costs	Medicine costs	Diagnosis costs	Transfusion costs	Total
API	227.19	42.69	745.16	28.29	143.08	226.58	5,581.44	947.98	27.52	346.28	5,877.39
RIV	472.41	48.48	1,053.4	65.31	114.88	350.38	1,515.54	1,105.24	35.68	477.44	3,072.13
DAB	338.21	75.05	1,165.71	41.40	101.26	309.75	1,428.48	715.78	47.91	486.07	3,926.16
LMWH/VKA	572.98	58.87	866.24	89.03	151.27	516.34	1,523.18	2056.08	28.91	278.44	4,325.38

API, apixaban; RIV, rivaroxaban; DAB, dabigatran; LMWH/VKA, low molecular weight heparin followed by vitamin K antagonist.

**TABLE 4:** Therapy efficacy and safety results.

	API (N = 50)	RIV (N = 110)	DAB (N = 21)	LMWH/VKA (N = 50)
Efficacy (%)				
Cure	22.0	2.7	4.8	6.0
Improvement	72.0	92.8	90.4	76.0
Therapy favorable	94.0	95.5	95.2	82.0
safety (%)				
Mortality	2.0	1.8	0.0	0.0
MB	0.0	0.0	0.0	0.0
CRNMB	2.0	2.7	4.8	10.0
VTE off-treatment	2.0	2.7	0.0	8.0
Therapy unfavorable	6.0	4.5	4.8	18.0

MB, major bleeding; CRNMB, clinically relevant non-major bleeding; VTE, venous thromboembolism. API, apixaban; RIV, rivaroxaban; DAB, dabigatran; LMWH/VKA, low molecular weight heparin followed by vitamin K antagonist.

Cost-effectiveness analysis results are shown in Table 5. The lowest ICER group was selected as the baseline group to calculate the ICER. In the base case, the RIV group resulted in a mean VTE attributable to 95% effective treatment. The API, DAB, and VKA have a negative ICER value (−187017.543, −284,674.922, and −9,283.339, respectively) and are absolutely inferior solutions.

**TABLE 5:** Base-case results.

	C	E	CER	ICER	Special
API	5,877.399	0.940	6,252.553	−187017.543	-
RIV	3,072.136	0.955	3,216.897	-	dominant
DAB	3,926.160	0.952	4,124.119	−284674.922	-
VKA	4,325.386	0.820	5,274.862	−9,283.339	-

QALY, quality-adjusted life year; ICER, incremental cost-effectiveness ratio; CER, the cost-effectiveness ratio; API, apixaban; RIV, rivaroxaban; DAB, dabigatran; LMWH/VKA, low molecular weight heparin followed by vitamin K antagonist.

### Markov Results

The cost-effectiveness values of the four regimens simulated by the Markov model after 30 years of treatment of VTE are shown in Table 6. The transition probability is shown in Supplementary Table S2. Compared with RIV, API was dominant in cost-effectiveness. The DAB and VKA strategy resulted in a slight increase in QALY (0.154 QALYs and 0.146 QALYs, respectively), and the corresponding increase in costs of $17031.885 and $122179.566 resulted in ICERs of $110577.872 per QALY and $836846.343per QALY, respectively. The incremental analysis results of DAB and VKA versus RIV exceeded the threshold range, which proved that DAB and VKA are not economical compared with RIV.

**TABLE 6:** Cost-effectiveness results of Markov model.

Strategy	Cost	Incremental cost	QALY	Incremental QALY	ICER	CER	Special
RIV	6,520.280	0	4.762	0.000	0	1,369.351	-
API	14,569.168	8,048.888	4.724	−0.037	−216176.977	3,083.846	Dominated
DAB	23,552.165	17,031.885	4.916	0.154	110,577.872	4,791.302	-
VKA	128,699.846	105,147.681	4.908	0.146	836,846.343	26,220.793	-

QALY, quality-adjusted life year; ICER, incremental cost-effectiveness ratio; CER, the cost-effectiveness ratio.

### Sensitivity Analysis

**FIGURE 2:**
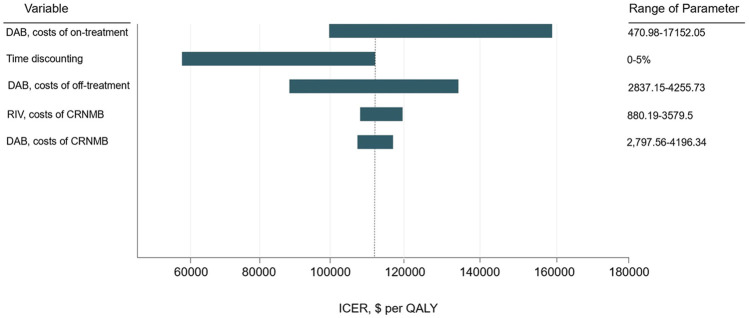
One-way sensitivity analysis tornado diagram.

One-way sensitivity analysis was performed using key parameters in the model, including cost and utility value, to assess the robustness of the model (Figure 2). The following factors including costs of the treatment of DAB, time discounting, the costs of the off-treatment using DAB, the costs of CRNMB of RIV, and the costs of MB using DAB have a significant influence on the result. One-way sensitivity analysis was conducted for the five most influential variables separately. RIV is still the most economically advantageous within the range of changes in sensitivity parameters. (Supplementary Table S3). Therefore, it can be inferred that changes in these two variables have no significant effect on the economic advantages of RIV.

**FIGURE 3:**
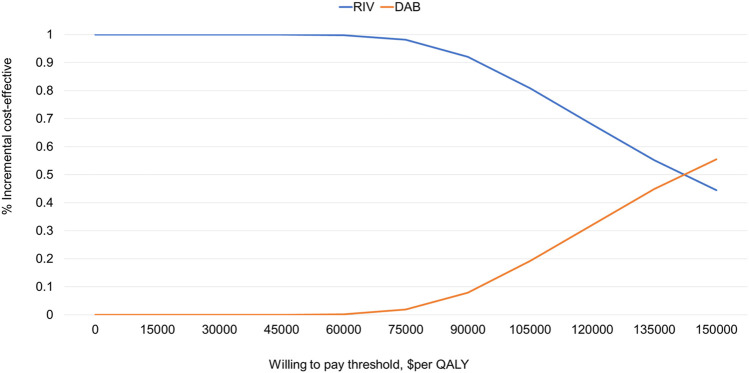
Cost-effectiveness acceptability curves.

Cost-effectiveness acceptability curves (CEAC) are shown in Figure 3. Within the threshold range selected, RIV has more economic benefits. RIV has a 100% probability of being cost-effective compared with other regimens when the willingness to pay (WTP) sis $10973 per QALY. When WTP exceeds US$148,000, DAB is more cost-effective than RIV.

**FIGURE 4:**
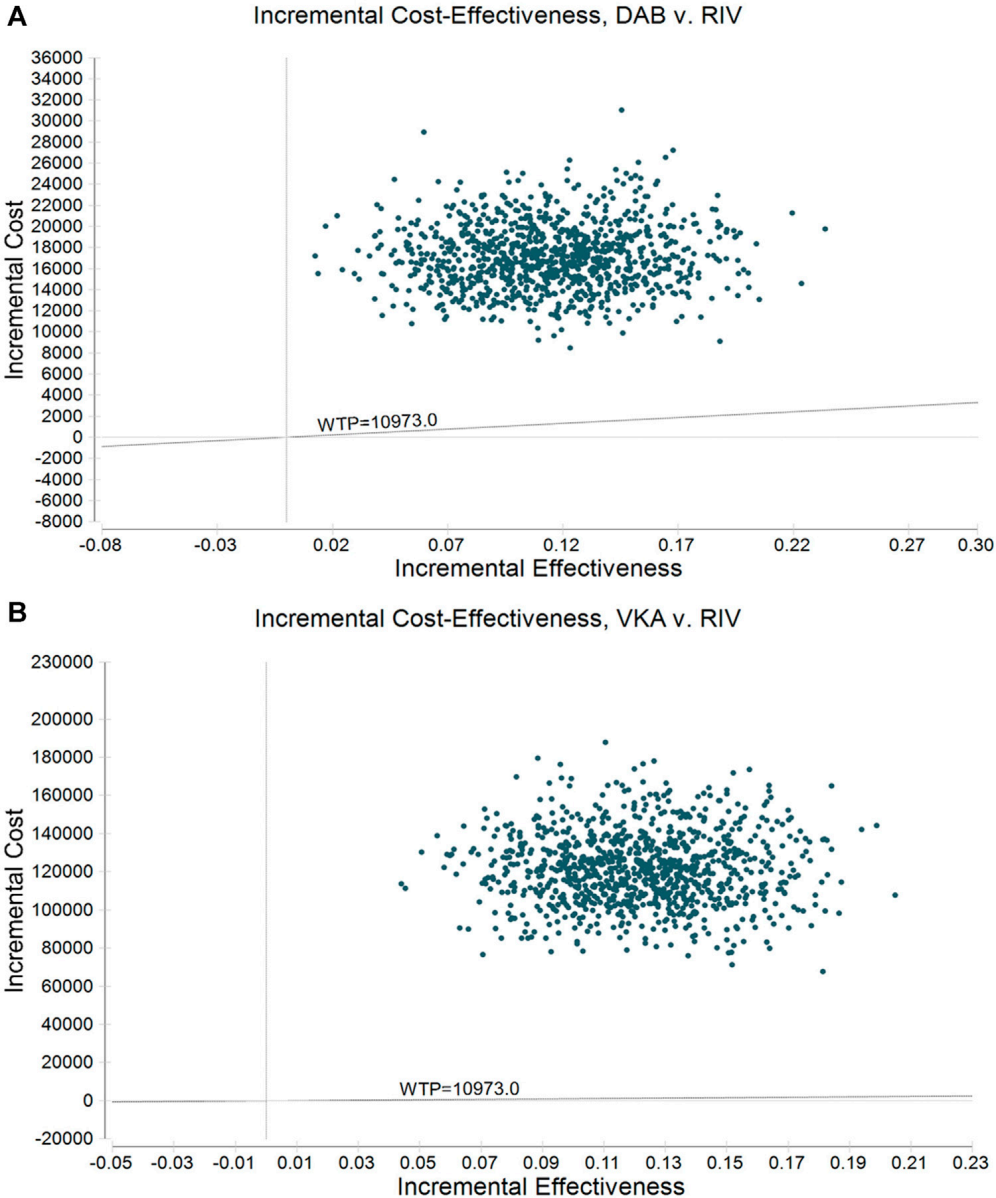
Incremental cost-effectiveness scatter plot of probabilistic sensitivity analysis. **(A)** Dabigatran vs. rivaroxaban; **(B)** LMWH + VKA vs. rivaroxaban.

One thousand iterations of Monte Carlo simulation methods to further explore the parameter uncertainty are presented in Figure 4. The scattered points were distributed more concentratedly inside the ellipse, indicating that the ICER analysis results of the scheme are relatively stable. The ICER for DAB versus RIV (Figures 4A) and VKA versus RIV (Figures 4B) was greater than $10973.0 per QALY for VTE patients.

## Discussion

This study conducted a pharmacoeconomic evaluation for VTE patients using DOACs and VKA standard therapy. RIV was dominant over the short-term hospitalization period. The Markov results we developed as part of the appraisal process verified this conclusion. We estimated that DAB was cost-effective compared with RIV when assuming a WTP threshold of $148000 per QALY in the exploratory analysis of the Markov model.

Our research has several advantages. Few economic evaluations have compared currently approved DOACs with LMWH + VKA for the treatment of VTE patients, especially in China. This study is, to our knowledge, the first research that compared the four therapies simultaneously. Two health outcomes, treatment effectiveness and QALY, were evaluated to determine the conclusion. The study complements the problem that RCT data are based on specific patient populations and specific study settings which may not truly reflect the actual health-care environment. Patients treated with RIV had the highest treatment favorable rate, which may be one of the reasons why RIV has the most economic advantage in clinical treatment. In addition, the model uncertainty was evaluated by using sensitivity analysis parameters.

Our research results have some differences and innovations from previous literature. In line with previous studies, Craig (Seaman et al., 2013) and Li Yang (Yang and Wu, 2020) examined the cost-effectiveness analysis of RIV for VTE treatment versus enoxaparin, which showed that RIV was a cost-effective therapy. However, Abdullah (Al Saleh et al., 2017) suggested that API was likely cost-effective for treatment durations of 3, 6, and 12 months versus DOAC. A study by Amin et al. (2016) found that this distinction probably stems from the fact that a vast majority of this study used EMR data, rather than using the parameters obtained by literature research like other studies. In addition, the definition of MB was slightly different in the respective literature. A study by Peter et al. (2016) divided massive bleeding into fatal MB and non-fatal intracranial bleeding.

The results of one-way sensitivity analysis found that the cost of on-treatment in DAB had the greatest impact on the model outcome. However, after calculating the range of upper and lower limits separately, RIV is still the most cost-effective, and the model is robust. The probability of choosing DAB gradually increases when the patient’s willingness-to-pay value exceeds $148,000. Especially, the results are meaningful for the Chinese health-care system, hospitals, and payers. In the case of the same curative effect, doctors can choose the most reasonable therapy according to the economic status of patients. Accounting for the increase in costs and ICER, the addition of VKA and DAB treatment was not an economically viable treatment option for VTE. Although lower price assumptions may not influence the overall cost-effectiveness results, further reductions such as social assistance or medical insurance may contribute to making DAB more affordable for VTE patients (Zhao et al., 2018).

Considering the disadvantage of API, the following facts may provide some explanations. API has a significant effect in reducing the risk of MB, and its safety and effectiveness are beyond those of similar drugs (Baber et al., 2014). But given the high price of API and the foreign patents that have not expired until 2023 (Tichy et al., 2021), the application scale of apixaban is still very rare in China (Yu et al., 2020). It is worth noting that with the launch of generic drugs in China, the reduction of the price of apixaban will lead to a more large-scale application, which probably makes it more economic.

### Limitations

First, the acquisition of transition probability parameters may have a certain impact on the research results. The parameters of the Markov model established in this study were derived from EMR and published literature. The model assumes that the transition probability was a fixed value. In contrast, the transition probability changes with time in the actual treatment process, which causes a certain bias in the model. Therefore, large-scale prospective studies should be used to reduce the resulting bias caused by the transition probability.

Second, another limitation in costs involves the process of collecting cost data. This study adopted the perspective of the health-care system for analysis. Although the complications may result in loss of work expenses and escort expenses for other members of the family, this part of the expenses is difficult to measure in actual follow-up, and it was not included in the study. Moreover, the patient’s mental loss due to illness was not included in the study, so the lack of indirect costs and hidden costs resulted in underestimation of the costs of the therapies to a certain extent. However, due to the small difference between the indirect costs and hidden costs of the four schemes, the impact on the results was little. In addition, this study conducted a sensitivity analysis on the cost of each health state of VTE and did not find any difference. Simultaneously, there are some uncertainties and limitations that arise from the use of EMR for cost-effectiveness analysis. For example, it cannot be determined that the patient was affected by other drugs during drug treatment. The confounding factors and bias of the data also need to be accurately analyzed.

Third, the health utility value obtained from the published literature could not accurately reflect the clinical effect on Chinese patients. Currently, there is no research on the utility value of VTE patients in China, so the utility value data caused by complications in this study refers to the assumptions of similar studies in the model. Due to differences in the level of economic development of different countries, there will be differences in health utility values (Locadia et al., 2004; Mccullagh et al., 2012). However, the sensitivity analysis results of this study suggest that this indicator has little effect on the results.

Fourth, although prolonging the time of anticoagulation therapy can reduce the recurrence rate of VTE by more than 80% (Couturaud et al., 2019; Wang et al., 2018), it does not reduce the risk of recurrence after patients stop using anticoagulant drugs. Since the Markov model simplifies the course of the disease, it will bias the results.

Fifth, in the results of patient data collection, 98% of the patient population treated with API has DVT, which can lead to the occurrence of confounding factors. On the one hand, physicians may adopt different treatment strategies for different disease types On the other hand, DVT patients are prone to post-thrombotic syndrome (Kahn, 2016), which is an important chronic complication of DVT and affects the results. The RIV group is quite older, is heavier, and has fewer females than all other groups. This would cause deviations because obesity, gender, and age can affect physicians’ choice of anticoagulant drugs (Mitchell and Conway, 2014; Loffredo et al., 2016; Perales et al., 2020).

Finally, this study did not conduct a subgroup analysis. In fact, in certain patient groups such as pregnant women, cancer patients, and elderly patients, the treatment of VTE is more challenging than the general population (Johannes et al., 2015; Boon et al., 2018). The anticoagulation treatment for specific populations needs to be carefully considered.

In summary, this study found that RIV is the most cost-effective treatment option in the treatment of VTE patients. Due to the limitations of the study, a large-scale prospective study of Chinese patients is still needed to confirm the results of economic evaluation.

## Conclusion

Short-term inpatient economic evaluation and Markov modeling suggest that relative to LMWH + VKA, DAB, and API, RIV could be considered as a more cost-effective or cost-saving long-term strategy for VTE patients in China. Nevertheless, further evidence is needed using data from large-scale studies.

## Data Availability

The datasets presented in this article are not readily available because the original data is confidential. Requests to access the datasets should be directed to K-XS, 18851101027@163.com
