# Supplementary material for: Cost-Effectiveness Analysis of Direct Oral Anticoagulants Versus Vitamin K Antagonists for Venous Thromboembolism in China
Source: Front Pharmacol. 2021 Oct 20;12:716224. doi: 10.3389/fphar.2021.716224 (PMC8563621; doi:10.3389/fphar.2021.716224)
Supplement: Supplementary file 1 [file DataSheet1.docx]

**Supplement**

**e Table 1:** The results of EMR data for hospitalization costs.

|  | Laboratory costs | Bed costs | Operation costs | Nursing costs | Radiation costs | Examination costs | Treatment costs | Medicine costs | Diagnosis costs | Transfusion costs | Total |
| --- | --- | --- | --- | --- | --- | --- | --- | --- | --- | --- | --- |
| API | 227.19 | 42.69 | 745.16 | 28.29 | 143.08 | 226.58 | 5581.44 | 947.98 | 27.52 | 346.28 | 5877.39 |
| RIV | 472.41 | 48.48 | 1053.4 | 65.31 | 114.88 | 350.38 | 1515.54 | 1105.24 | 35.68 | 477.44 | 3072.2 |
| DAB | 338.21 | 75.05 | 1165.71 | 41.40 | 101.26 | 309.75 | 1428.48 | 715.78 | 47.91 | 486.07 | 3926.19 |
| VKA | 572.98 | 58.87 | 866.24 | 89.03 | 151.27 | 516.34 | 1523.18 | 2056.08 | 28.91 | 278.44 | 4325.34 |

**e Table 2:** Cost of drugs

|  | Manufacturer information | Dosage | Cost/specification | Usage and dosage direction | Price change |
| --- | --- | --- | --- | --- | --- |
| API | Bristol Myers Squibb Srl | Tablet | 39.47/2.5mg | 10mg BID for 7days, followed by 5 mg BID | 0.97-47.35 |
| RIV | Bayer (Schweiz) AG Delpharm Gaillard | Tablet | 27.75/15 mg;  34.6/20 mg | 15mg BID for 21 days, followed by 20mg daily | 15mg:27.75-99.37  20mg: 34.59-110.14 |
| DAB | Boehringer Ingelheim (Schweiz) GmbH | Capsule | 20.00/1ml:5000iu；  21.82/150mg | LMWH BID for 5 days, followed by 150mg BID | LMWH:8.50-30.37  DAB:21.38-21.83 |
| VKA | SCIPROGEN BIO-PHARMACEUTICAL | Injection/Tablet | 20.00/1ml:5000iu；  0.18/2.5mg | LMWH BID for 4days, followed by 5mg daily | LMWH: 8.50-30.37  VKA: 0.17-0.23 |

**e Table 3:** Results of single factor sensitivity analysis

|  | strategy | IncrEff | IncrCost | IC/IE | Avg CE | Dominance |
| --- | --- | --- | --- | --- | --- | --- |
| TX=MIN |  |  |  |  |  |  |
|  | RIV | 0 | 0 | 0 | 1498.09 | dominant |
|  | API | -0.15 | 12430.71 | -81351.25 | 3371.95 |  |
|  | DAB | 0.44 | 25461.29 | 57858.36 | 4873.07 |  |
|  | VKA | -0.23 | 151117.74 | -663706.51 | 26249.98 |  |
| TX=MAX |  |  |  |  |  |  |
|  | RIV | 0 | 0 | 0 | 1359.91 | dominant |
|  | API | -0.13 | 6906.18 | -533365.56 | 3060.47 |  |
|  | DAB | 0.09 | 14365.14 | 149627.17 | 4764.97 |  |
|  | VKA | 0.01 | 89097.11 | 7733981.31 | 26006.30 |  |
| DAB_off-treatment =MIN |  |  |  |  |  |  |
|  | RIV | 0.00 | 0 | 0 | 1369.35 | dominant |
|  | API | -0.04 | 8048.89 | -216176.98 | 3083.85 |  |
|  | DAB | 0.15 | 15136.11 | 98269.75 | 4405.63 |  |
|  | VKA | -0.00 | 107043.45 | -14670844.09 | 26220.79 |  |
| DAB_off-treatment=MAX |  |  |  |  |  |  |
|  | RIV | 0 | 6520.28 | 0 | 1369.35 | dominant |
|  | API | -0.04 | 14569.17 | -216176.98 | 3083.84 |  |
|  | DAB | 0.15 | 30808.80 | 157690.91 | 5517.36 |  |
|  | VKA | -0.00 | 128699.84 | -13416459.86 | 26220.79 |  |
| DAB_on-treatment=MIN |  |  |  |  |  |  |
|  | RIV | 0 | 0 | 0 | 1369.35 | dominant |
|  | API | -0.04 | 8048.89 | -216176.98 | 3083.84 |  |
|  | DAB | 0.15 | 13462.86 | 87406.35 | 4065.24 |  |
|  | VAK | -0.00 | 108716.70 | -14900171.23 | 26220.79 |  |
| DAB_on-treatment=MAX |  |  |  |  |  |  |
|  | RIV | 0 | 0 | 0 | 1369.35 | dominant |
|  | API | -0.03 | 8048.88 | -216176.97 | 3083.84 |  |
|  | VAK | 0.15 | 122179.56 | 832683.97 | 26220.79 |  |
|  | DAB | 0.00 | 2018414.68 | 276633891.95 | 436795.25 |  |
|  |  |  |  |  |  |  |
| DAB_CRNMB=MIN |  |  |  |  |  |  |
|  | RIV | 0 | 0 | 0 | 1369.35 | dominant |
|  | API | -0.04 | 8048.88 | -216176.98 | 3083.85 |  |
|  | DAB | 0.15 | 17031.88 | 110577.87 | 4791.30 |  |
|  | VKA | -0.00 | 105147.68 | -14411018.98 | 26220.79 |  |
| DAB_CRNMB=MAX |  |  |  |  |  |  |
|  | RIV | 0 | 0 | 0 | 1369.35 | dominant |
|  | API | -0.03 | 8048.89 | -213745.67 | 3083.85 |  |
|  | DAB | 0.15 | 24688.75 | 347468.43 | 145467.69 |  |
|  | VKA | -0.00 | 105147.68 | -14411018.98 | 26220.79 |  |
| RIV_CRNMB=MIN |  |  |  |  |  |  |
|  | RIV | 0 | 0 | 0 | 1129.07 | dominant |
|  | API | -0.04 | 9193.01 | -246905.81 | 3083.85 |  |
|  | DAB | 0.15 | 18176.01 | 118005.996 | 4791.30 |  |
|  | VKA | -0.01 | 105147.68 | -14411018.98 | 26220.79 |  |
| RIV_CRNMB=MAX |  |  |  |  |  |  |
|  | RIV | 0 | 0 | 0 | 1491.90 | dominant |
|  | API | -0.03 | 7465.33 | -200503.88 | 3083.85 |  |
|  | DAB | 0.15 | 16448.33 | 106789.20 | 4791.30 |  |
|  | VKA | -0.0073 | 105147.68 | -14411018.98 | 26220.79 |  |
